# Early supported discharge for older adults admitted to hospital after orthopaedic surgery: a systematic review and meta-analysis

**DOI:** 10.1186/s12877-024-04775-y

**Published:** 2024-02-09

**Authors:** Susan Williams, Cliona O’Riordan, Ann-Marie Morrissey, Rose Galvin, Anne Griffin

**Affiliations:** 1 School of Allied Health, University of Limerick, Limerick, Ireland; 2 University Hospital Limerick, Dooradoyle, Limerick, Ireland

**Keywords:** Early supported discharge, Older adults, Hospitalised, Systematic review, Orthopaedic

## Abstract

**Background:**

Early supported discharge (ESD) aims to link acute and community care, allowing hospital inpatients to return home, continuing to receive the necessary input from healthcare professionals that they would otherwise receive in hospital. Existing literature demonstrates the concept having a reduced length of stay in stroke inpatients and medical older adults. This systematic review aims to explore the totality of evidence for the use of ESD in older adults hospitalised with orthopaedic complaints.

**Methods:**

A literature search of Cochrane Central Register of Controlled Trials in the Cochrane Library (CENTRAL), EMBASE, CINAHL and MEDLINE in EBSCO was carried out on January 10th, 2024. Randomised controlled trials or quasi-randomised controlled trials were the study designs included. For quality assessment, The Cochrane Risk of Bias Tool 2.0 was used and GRADE was applied to evaluate the certainty of evidence. Acute hospital length of stay was the primary outcome. Secondary outcomes included the numbers of fallers and function. A pooled meta-analysis was conducted using RevMan software 5.4.1.

**Results:**

Seven studies with a population of older adults post orthopaedic surgery met inclusion criteria, with five studies included in the meta-analysis. Study quality was predominantly of a high risk of bias. Statistically significant effects favouring ESD interventions were only seen in terms of length of stay (FEM, MD = -5.57, 95% CI -7.07 to -4.08, I^2^ = 0%). No statistically significant effects favouring ESD interventions were established in secondary outcomes.

**Conclusion:**

In the older adult population with orthopaedic complaints, ESD can have a statistically significant impact in reducing hospital length of stay. This review identifies an insufficient existing evidence base to establish the key benefits of ESD for this population group. There is a need for further higher quality research in the area, with standardised interventions and outcome measures used.

**Supplementary Information:**

The online version contains supplementary material available at 10.1186/s12877-024-04775-y.

## Introduction

Global population projections indicate that the number of adults aged ≥ 65 years (older adults) will more than double from one billion in 2019 to 2.1 billion in 2050 [1]. As the world’s population continues to live longer, the prevalence of older adults presenting to acute hospitals with multiple co-morbidities requiring more complex management will continue to increase [2]. This changing demographic to an ageing population will augment the demands older adults place on health services as the largest users. Planning and adapting a health service for this anticipated increase in service demand is a priority [3].

Older adults are known to be the greatest users of health services, with over half of those presenting to emergency departments being admitted for inpatient care [4]. Older adults are more likely to experience a prolonged length of stay (LoS), [5] and are at a higher risk of experiencing adverse events, such as hospital associated functional decline, hospital acquired infection and reduced mobility [6]. Post orthopaedic surgery, older adults are at an increased risk of post operative complications such as delirium, increased levels of dependency and an increased risk of mortality, which can result in more complex care needs at discharge due to a reduction in patient independence and a subsequent longer hospital LoS [7, 8]. Geriatric syndromes including reduced mobility, incontinence and a change in cognitive status are the greatest predictors of post-operative complications [9].

Discharge planning interventions are used in acute hospitals to facilitate a patient’s progression back to their community or home setting once medically well [10, 11]. Early supported discharge (ESD) aims to link acute and community care, by providing older adults with input in their own home by healthcare professionals, allowing them to return home sooner than otherwise would be possible with community care [12]. Langhorne and Baylan [12] carried out a Cochrane review of 17 randomised control trials (RCTs) providing ESD interventions in acute stroke care. In the intervention group, an average reduction of six days (MD = -5.5; 95% CI ‐3 to ‐8 days; *P* < 0.0001) in hospital LoS was found. In older adults admitted to hospital for medical complaints, a systematic review and meta-analysis carried out by Williams, Morrissey [13] found statistically significant effects favouring ESD interventions in terms of patient LoS (REM, MD = -6.04, 95% CI -9.76 to -2.32, I^2^ = 90%, *P* = 0.001).

To the extent of the authors’ knowledge, the existing evidence base regarding the effectiveness of an ESD intervention for older adults admitted to hospital for orthopaedic complaints has not been explored. The aim of this paper is to systematically review the totality of evidence exploring the effectiveness of ESD on process and clinical outcomes in older adults admitted to hospital for orthopaedic complaints.

## Methods

### Study design

A systematic review and meta-analysis was carried out. This review was registered with PROSPERO, ID: CRD42023420524. The Preferred Reporting Items for Systematic Reviews and Meta-Analysis (PRISMA) guidelines were followed [14]. The Cochrane Handbook for Systematic Reviews of Interventions was adhered to as appropriate [15]. A protocol was not developed for this systematic review. The methods for this systematic review are based upon those carried out by Williams, Morrissey [13] in their systematic review investigating ESD for older medical inpatients.

### Study identification

Searches were carried out in the following databases on 10th January 2024: CENTRAL, MEDLINE in Ebsco, CINAHL in Ebsco and EMBASE. The search strategy consisted of four sections broadly covering the topics of ESD, older adults, orthopaedic care and RCT study design. MeSH terms and appropriate controlled vocabularies and associated keywords were used. Search strategies were based on those carried out by Williams, Morrissey [13] in their previous systematic review of ESD in older medical inpatients. Grey and unpublished literature was not included in the search strategy. Full search strategies can be seen in Additional File 1. Studies were limited from the year 1997 onwards as this was when ESD was first introduced for stroke care [16, 17].

Inclusion criteria was as follows:

Population - Studies were included if > 50% of the study population were older adults (aged ≥ 65 years) who were admitted to the acute care setting for orthopaedic complaints.

Intervention - Studies were required to provide an ESD intervention, described as interventions aimed to accelerate patient discharge from hospital once medically stable, and providing patients with the necessary input in the community at the same level of intensity and resources they would receive while in the inpatient setting [12]. These interventions are typically provided within 24–48 h of hospital discharge by a multidisciplinary team (MDT) within the patient’s own home, for a period of six-eight weeks. Patients receive the home-based rehabilitation at a frequency and intensity deemed appropriate by the patient’s treating clinicians.

Control - usual care as described by study authors, other non-ESD interventions such as transfer to rehabilitation facilities or continuing MDT input in the inpatient setting, or an absence of ESD interventions.

Outcome - the primary outcome measure was acute hospital LoS. Secondary outcomes were focused on patient and process outcomes inclusive of function, quality of life, incidence of falls, use of primary care services and hospital representations and readmissions.

RCTs (including cluster trials) and quasi-RCTs were included in this systematic review. Studies were excluded if they did not meet all the above inclusion criteria.

### Study selection

Results from all databases were placed in a master Endnote library [18]. The primary author (SW) manually screened the studies for duplicates. Studies were screened by title and abstract in Endnote against the inclusion criteria by SW. If further information was sought, authors were contacted by SW.

Studies in the unsure group initially underwent an independent review by a second author (AG) by title/abstract. A full text review was carried out by AG for any remaining unsure studies. Rayyan software was used to facilitate this. If an agreement could not be met, a third author was consulted (RG). Three authors (SW, AG and RG) reviewed all studies in the final relevant group to ensure they met the inclusion criteria.

### Quality assessment

The Cochrane Risk of Bias Tool 2.0 was used to quality assess the included studies [19]. This tool assesses risk of bias across five domains - the randomisation process, deviations from intended interventions, missing outcome data, measurement of the outcome and selection of the reported result. Quality assessment was carried out independently by SW and AG. Any discrepancies were discussed, and a third author (RG) was consulted for any disagreements. Study protocols were used as available to guide the quality assessment. The Grading of Recommendations, Assessment, Development and Evaluations (GRADE) framework was used to assess the certainty of evidence independently by SW and RG [20].

### Data extraction & statistical analysis

Descriptive data (author, year, country, method, population, intervention, control, outcomes measured) were independently extracted by SW. The authors of included studies were contacted if data were missing.

The Cochrane Review Manager software (RevMan, V.5.4) was used to perform the statistical analysis. If the mean and standard deviation (SD) was not available, the inter-quartile range (IRQ) was multiplied by 0.75 and the difference in the range was multiplied by 0.25 [21]. In reporting follow-up data, if outcomes were not reported at the same timepoints post intervention, the timepoints that aligned most closely were used, typically at three- and 12-month follow-up. In studies that assessed the same outcome but used different scales, the treatment effect was determined using the standardized mean difference (SMD). In studies that measured the same outcome using the same scales, the mean difference (MD) was used. For all outcomes, the denominator in each group was considered as the number of participants allocated to that group at baseline.

We assessed known variability across the studies by examining the characteristic of the population of interest, the content and duration of the ESD intervention, outcomes examined and the duration of follow up. Where minimal or no study heterogeneity was evident, a meta-analysis was performed. Heterogeneity was also explored by visually inspecting the forest plots and the associated Chi^2^ and I^2^ statistics. As stringent cut-offs for interpreting I^2^ are no longer recommended, we applied the approximate guide by Deeks, Higgins [15] when interpreting the I^2^ statistic. Where there was evidence of significant heterogeneity, we present the more conservative random-effects model (REM) outcome.

In the absence of data available for pooled analysis, a narrative description was provided. The narrative synthesis was completed by developing a preliminary synthesis to describe patterns of results in terms of direction and size, as appropriate. Factors which may contribute to the differences in direction and size were then considered to explore the relationships between the data. Finally, conclusions were drawn based upon these considerations to determine the strength of the evidence.

## Results

### General overview and search strategy

**Fig. 1:**
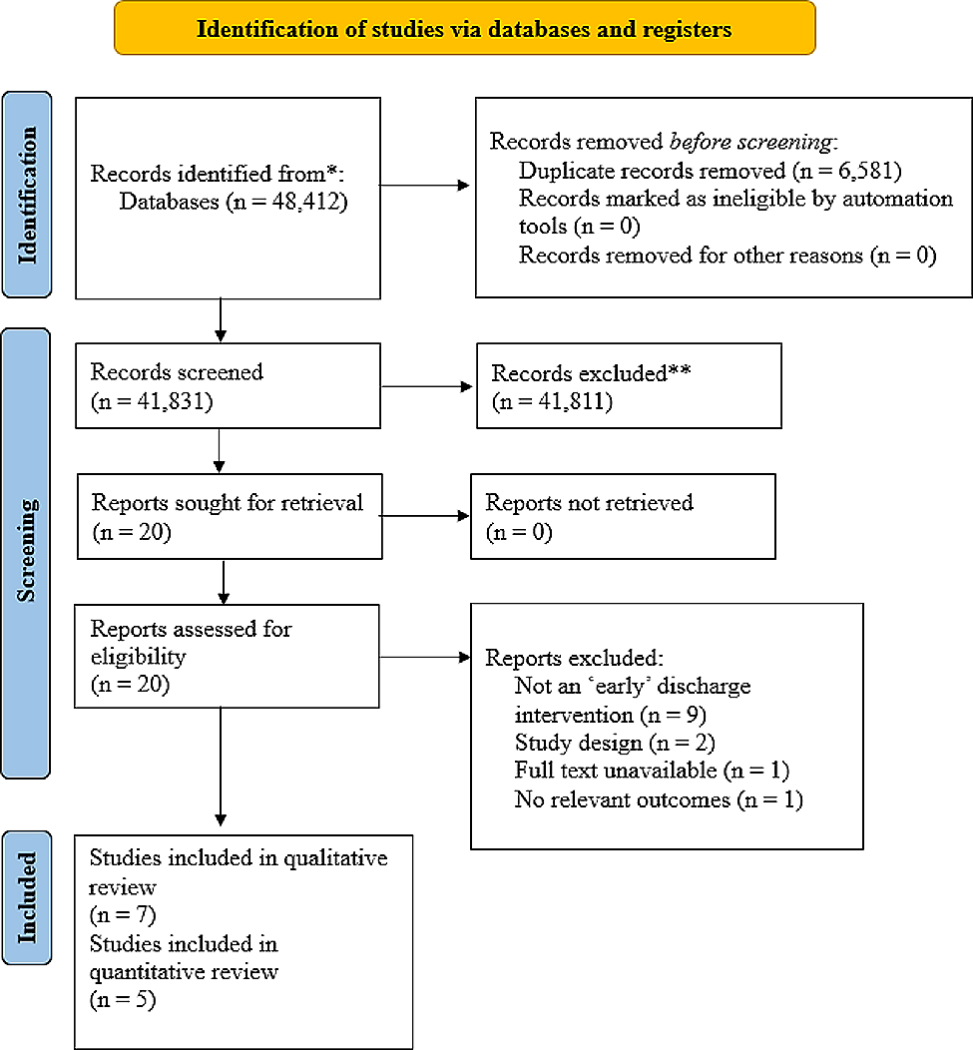
PRISMA flow diagram

Initial database searching identified 48,412 studies. 20 studies were screened for full text review from which seven papers were included in the quantitative synthesis. Five of these reported data suitable for meta-analysis. The PRISMA flow diagram summarizing the identification and selection of included studies can be seen in Fig. 1.

The characteristics of included studies are summarized in Table 1. All included studies were RCTs, except for Closa, Mas [22] which was a quasi-RCT. Along with this, the included studies comprised of three RCTs carried out by Crotty, Whitehead [23], Parsons, Parsons [24] and Karlsson, Berggren [25]. A further three studies reported secondary data from these studies [26–28]. All studies included a population of those aged ≥ 65 years of whom more than 50% received a surgical intervention post hip fracture. All patients were deemed medically fit for discharge but required further MDT intervention.

All interventions were MDT based, but the composition of the MDT varied slightly per study. All included physiotherapists and occupational therapists, with Closa, Mas [22], Parsons, Parsons [24] and Karlsson, Berggren [25] all incorporating nursing staff. Geriatrician support was provided by all interventions excluding Closa, Mas [22], with all except for Karlsson, Berggren [25] having weekly MDT case conferences. Only Crotty, Whitehead [23] included a speech and language therapist and assigned a team coordinator for each patient. Crotty, Whitehead [23] was the only study to include a social worker, although social work was available on consult for Karlsson, Berggren [25].

The intensity of the intervention varied by study. Crotty, Whitehead [23] did not specify the minimum/maximum length or frequency of the intervention provided, reporting that the intervention was tailored to the patients individual needs. Karlsson, Berggren [25] provided the intervention for a maximum of ten weeks, although the frequency was not reported. However, Parsons, Parsons [24] provided the intervention seven days a week comprising of up to four visits per day for a total of six weeks. Closa, Mas [22] provided therapy intervention for up to five days/week for 35–45 min per session and up to seven nursing visits/week but did not specify the overall duration of the intervention. The control group for all studies was usual care, which comprised of inpatient hospital rehabilitation and onward referral to existing community services as appropriate.

All studies followed their participants up to 12 months post intervention. LoS was the most commonly assessed outcome [22–24, 28]. Other outcomes assessed included function, re-admissions, and falls.

### Methodological quality

Study quality under the various tools of the Cochrane Risk of Bias Tool 2.0 can be seen in Additional File 3. There was a large variation in results across the included studies. Crotty, Whitehead [23] were considered as low risk of bias across all domains. On the contrary, Karlsson, Berggren [25] and their subsequent papers Berggren, Karlsson [28] and Karlsson, Lindelöf [27] were deemed to be of high risk of bias overall due to the measurement of their outcomes. Closa, Mas [22] was also deemed to be of high risk of bias due to their randomisation process. Additional File 4 summarises the certainty of the evidence for outcomes included in the meta-analyses using the GRADE framework [20]. All outcomes were deemed to be of very low to low certainty, except for LoS which was deemed to be of moderate certainty. Although publication bias is deemed a part of the GRADE assessment, the Cochrane Handbook for Systematic Reviews of Interventions advises publication bias assessed using funnel plot asymmetry should only be used when there are at least 10 studies included in the meta-analysis [15]. Therefore, publication bias was not assessed.

**Table 1:** Characteristics of Included Studies

Author	Year	Country	Methods	Population	Intervention	Control	Outcomes Measured
Closa et al.	2017	Spain	Quasi-RCT On discharge from the acute orthopaedic surgery/traumatology unit, patients who met the inclusion criteria were admitted to the HHU if an effective primary caregiver was willing to accept responsibility for the home-based program and the patient agreed to hospitalization at home. Otherwise, patients were admitted to the hospital-based post-acute GRU	Aged > 65 years, attended by an acute orthopaedic surgery/ traumatology unit (at the emergency department or at ward) after a fracture or elective arthroplasty with good orthopaedic prognosis, decline in functional status in relation to baseline characteristics susceptible to rehabilitative treatment and clinical status sufficiently stable to enable active participation in a rehabilitation program.	Hospital-at-home integrated care unit (HHU) (*n* = 91) - all patients underwent a CGA to develop a care plan focusing on cardiorespiratory function and nutritional status, detection of delirium and cognitive impairment, treatment of pain, and prevention of pressure ulcers. Nursing visits were limited to 7 per week; physiotherapy and occupational therapy sessions were limited to 5 per week. Each therapy visit lasted 35 to 45 min.	Geriatric rehabilitation unit (GRU) (*n* = 276) - received the same geriatric assessment and nursing care, as well as physiotherapy and occupational therapy following hospital ward guidelines (maximum duration 1 h per session, limited to 5 sessions per week)	BI Heinemann Index Direct cost of care LoS
Crotty et al.	2002	Australia	RCT, participants from three hospitals, computer generated randomisation by a hospital pharmacist who had no other involvement in the study	Aged ≥ 65 years living in the Adelaide Southern Metropolitan Region treated surgically for a hip fracture, medically stable, had adequate physical and mental capacity to participate in a rehabilitation programme, expected to return home after discharge from hospital and had a home environment suitable for rehabilitation	Acceleration discharge and home-based rehabilitation (*n* = 34) - initial assessment by the study co-ordinator who visited their home environment and organized any modifications, installation of equipment or assistive aids prior to discharge. GPs were contacted and asked to consent to their patient participating in the programme. Participants were discharged from acute care within 48 h of randomization and promptly visited by therapists from the home rehabilitation team including a team co-ordinator, a physiotherapist, an occupational therapist, a speech pathologist, a social worker and a therapy aid, who negotiated realistic, short-term and measurable treatment goals with both the participant and their carer. Therapy frequency was tailored to the needs and rate of progress of individual participants, and structured practice sessions were encouraged between visits. Progress was reviewed at weekly case conferences attended by all staff and a specialist in rehabilitation medicine or a geriatrician.	Conventional care (*n* = 32) - routine hospital care and rehabilitation in hospital; inpatient services and the development of care pathways and discharge planning	Four months post: TUG MBI Balance Confidence Scale FES BBS London Handicap Scale Falls Hospital re-admissions Patient and carer satisfaction SF-36 (patients and carers) Use of community services e.g. home help, district nursing CSI
Crotty et al.	2003	Australia	As per Crotty et al. (2002)	As per Crotty et al. (2002)	As per Crotty et al. (2002)	As per Crotty et al. (2002)	12 months post: TUG MBI SF-36 MBI CSI Changes in Residence Mortality
Karlsson et al.	2016	Sweden	RCT, using sequentially numbered lots in opaque, sealed envelopes drawn by a nurse at the ward, not involved in the study. The randomization was stratified into 2 categories according to type of housing (ordinary housing or residential care facilities) and type of fracture (cervical or trochanteric).	Aged ≥ 70 years post-acute hip fracture surgery (cervical or trochanteric fracture) and living in the municipality of Umeå in ordinary housing or in residential care facilities. Pathological or in hospital fractures were excluded. Patients were considered to have no medical obstacles, could manage basic transfers, and had the care they required at home	Conventional geriatric care and rehabilitation with Geriatric Interdisciplinary Home Rehabilitation (GIHR) after discharge (*n* = 107) - treated according to the multifactorial rehabilitation programme, including CGA with focus on detection, prevention, and treatment of postoperative complications. The GIHR team included a nurse, an occupational therapist, and two physiotherapists who visited the participants regularly. A geriatrician was medically responsible, and a social worker and a dietician could be consulted when necessary. Rehabilitation was individually designed according to the participants’ own goals. During the first days after discharge, all participants received nearly daily home visits from someone in the GIHR team and later according to the participants’ needs. The maximum duration in GIHR was 10 weeks.	Convention care and geriatric ward rehabilitation (*n* = 98) - interdisciplinary rehabilitation using CGA with regular meetings and individual rehabilitation plan	At 3 and 12 months: Walking ability indoors Walking ability outdoors Use of a gait aid Self-chosen gait speed Maximum gait speed LoS from surgery to hospital discharge LoS from admission to geriatric ward until discharge ready date LoS from admission to geriatric ward until discharge Rehabilitation received after discharge
Karlsson et al.	2020	Sweden	As per Karlsson et al. (2016)	As per Karlsson et al. (2016)	As per Karlsson et al. (2016)	As per Karlsson et al. (2016)	At 3 and 12 months: BI ADL Staircase
Berggren et al.	2019	Sweden	As per Karlsson et al. (2016)	As per Karlsson et al. (2016)	As per Karlsson et al. (2016)	As per Karlsson et al. (2016)	Complications at 3 & 12 months: Infection Cardiovascular event DVT PE Stroke Gastric ulcer Decubital ulcer Fallers Falls Additional fracture Luxation Reoperation Deceased Delirium Days with delirium LoS Re-admissions Days in hospital after discharge
Parsons et al.	2019	New Zealand	RCT, participants were randomized using a computer-generated randomization sequence	Older adults who had suffered an injury that required hospital admission and subsequent rehabilitation as well as meeting the START inclusion criteria: ≥ 65 years of age; in hospital at time of referral and did not require ongoing acute hospital-based treatment (in the judgment of the consultant geriatrician); consented to being treated at home; and agreed with the objectives set by the referring inter-disciplinary team. Following assessment by the referring team, the participant was considered to have potential for partial or complete recovery with suitable home rehabilitation within 6 weeks; was able to stand and transfer with 1person (with or without the help of a resident carer); had a recent injury and was at a borderline level of function with an associated reduction in activities of daily living (ADL) and/or instrumental ADL (IADL); and who without input from the team was considered likely to fail to recuperate full potential of functional recovery or was likely to fail to manage satisfactorily at home despite conventional community support and, therefore, would be at risk of hospital re-admission or institutionalization	Early SDT Intervention (START) (*n* = 201) - consisted of healthcare assistants (HCAs), registered nurses (RNs) and allied health (physiotherapists and occupational therapists). Consultant geriatricians provide input through weekly case conferences. HCAs provide up to 4 visits a day, 7 days a week. Patients are limited to 6 weeks attendance, though the team on an exception basis may choose to extend this to maximize potential recovery. The rehabilitation program is developed jointly by RNs and allied health professionals and progress is discussed within the team. Interdisciplinary practice is core to the delivery of the model; HCAs, RNs, and allied health meet daily to discuss patient progress and find solutions for problems as they arise.	Usual care (*n* = 202) - discharge planning from the hospital to their place of residence and subsequent community-based services as required. Community-based services could include allied health, district nursing, and home care.	LoS Re-admissions Time in hospital in the following 12 months Inpatient costs - index admission Inpatient costs - re-admissions Community care costs in the following 12 months interRAI Functional Assessment

### Primary outcome

**Fig. 2:**
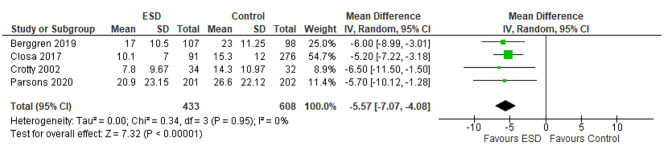
Forest plot for LoS

The primary outcome was LoS, reported as the number of days spent as an inpatient during the acute hospital stay. Four studies reported data suitable for analysis [22–24, 28]. As can be seen in Fig. 2, there were statistically significant effects favouring ESD for LoS between the intervention (*n* = 433) and control groups (*n* = 608), (|REM, MD = -, 95% CI -7.07 TO -4.08, I^2^ = 0%, *P* = 0.95). A moderate certainty of evidence was seen in the GRADE assessment for LoS.

### Secondary outcomes

Secondary outcomes measured included re-admissions, carer self-reported quality of life (QoL), gait and walking ability, post-operative complications, use of community services, inpatient and community financial costs, function, and number of fallers. Only data collected for function and the number of fallers was suitable for meta-analysis. The variation in how data was reported did not allow for meta-analysis across numerous outcomes. Corresponding authors for studies were contacted to gather data in alternative formats for meta-analysis, however this was not available.

Re-admission rates were similar in the intervention and control groups when measured at four and twelve months respectively [23, 28]. A very low certainty of evidence was found in the GRADE assessment for re-admissions. The number of days patients spent in hospital after discharge was measured at the same timepoints [23, 24, 28].

Balance was assessed at four months using the Berg Balance Scale by Crotty, Whitehead [23], with results favouring the intervention group, although these were not considered statistically significant. Carer self-reported QoL was measured using the Short Form-36, with statistically significant effects favouring the intervention group seen at twelve months (MD = 0.01, 95% CI -5.0 to 0.0) [23, 26]. As per the GRADE assessment, there was a very low certainty of evidence for carer QoL. Gait and walking ability was assessed using non-standardized outcome measures such as patients using a gait aid indoors and outdoors and gait speed [25]. Results were similar in both intervention and control groups at three and twelve month follow up. Berggren, Karlsson [28] measured complications including infection, cardiovascular events and deep vein thrombosis/pulmonary embolism with no differences between the intervention and control groups at twelve months. The use of community services was measured by one study, with 63% of the intervention group receiving input versus 77% of the control group, although this is not considered statistically significant [23].

Inpatient and community financial costs were measured by Closa, Mas [22] and Parsons, Parsons [24]. For Parsons, Parsons [24] the mean inpatient cost per patient in the intervention group was NZ $24,166 compared to NZ $26,161 in the control group. Re-admission costs per patient were NZ $5,976 in the intervention group and NZ $10,195 for the control group. Community costs over twelve months were also less for the intervention group at NZ $34,291 per patient, and NZ $37,167 in the control group. Meanwhile, Closa, Mas [22] reported the mean overall cost of care per patient in the intervention group to be €7,120 versus €12,149 in their control group.

Function was reported by four studies, with data reported by Closa, Mas [22], Crotty, Whitehead [26] and Karlsson, Lindelöf [27] used for the meta-analysis as this data was reported 12 months post intervention. Additional File 5 demonstrates no statistically significant effects were seen favouring ESD interventions (*n* = 232) when compared to the control group (*n* = 406) (REM, MD = -0.02, 95% CI -1.13 to 1.08, I^2^ = 0%, *P* = 0.93). A low certainty of evidence was identified in the GRADE assessment.

The number of fallers per population was reported by Berggren, Karlsson [28] and Crotty, Whitehead [23]. As can be seen in Additional File 5, no statistically significant effects were seen in the ESD group (*n* = 140) when compared to the control group (*n* = 125) (REM, RR = 1.08, 95% CI 0.79 to 1.49, I^2^ = 0%, *P* = 0.64), with a very low certainty of evidence as per the GRADE assessment. Crotty, Whitehead [23] measured this outcome for four months post intervention, whereas Berggren, Karlsson [28] followed their participants for 12 months post intervention.

## Discussion

### Statement of key findings

This systematic review evaluated the totality of evidence with respect to ESD interventions in older adults admitted to hospital for orthopaedic complaints. All studies were focused on the traumatic hip fracture population, with one study including those post elective arthroplasty, and one including those with fractures other than hip fractures. There was a statistically significant effect of ESD interventions found in LoS in this population. No statistically significant effects favouring ESD interventions were established in function or number of fallers post intervention. Meta-analysis was not possible for secondary outcomes: re-admissions, carer self-reported QoL, gait and walking ability, post-operative complications, use of community services, inpatient and community financial costs. Except for LoS, there was a low to very low certainty of evidence across outcomes assessed.

### Results in context of current literature

Our results are in keeping with a Cochrane review of ESD for the stroke population discussed previously by Langhorne and Baylan [12], demonstrating a statistically significant reduction in LoS, with no adverse outcomes in terms of function or number of fallers when compared to usual care. A systematic review and meta-analysis by Williams, Morrissey [13] also demonstrates a statistically significant reduction in LoS for older adults admitted to hospital with medical complaints.

All studies included in this review investigated ESD in an older adult population post-surgical intervention for traumatic hip fracture except for Closa, Mas [22] who also included hip/knee arthroplasty and pelvic fractures as approximately 40% of their total population. Voeten, Krijnen [29] carried out a systematic review of 16 studies to identify quality indicators for hip fracture care. Nine audits and five guidelines were included in the review. The authors identified nine key quality indicators for consideration in future research, including the return of patients to their place of residence within a specific timeframe. A systematic review and meta-analysis of 22 studies carried out by Lee and Lee [30] found that home based rehabilitation was as effective in terms of strength, balance gait and QoL in the hip fracture population. The authors identified the need for more home-based rehabilitation models of care for this population group, which ESD aligns to. Along with this, recent evidence would suggest early rehabilitation leads to better patient outcomes [31].

Leland, Lepore [32] investigated the opinions of physiotherapists and occupational therapists through focus groups of their perceptions on quality hip fracture care. Ninety-nine practitioners were involved across 13 semi-structured groups. Among the themes identified were facilitating a safe and early discharge home for patients and ensuring that patients avoid re-admission by equipping the patient and their caregivers with the knowledge and skills to manage at home after discharge. In the stroke population, research suggests that involving family members in ESD interventions can allow for patients and their caregivers to feel more prepared for managing at home, despite having no impact on the patients’ functional outcomes [33]. None of the studies included in this systematic review involved family members in their interventions, despite the benefits suggested.

In an older population who undergo surgical procedures, Colburn, Mohanty [34] developed guidelines for immediate post-operative care, consulting geriatricians, anaesthetists and surgeons. The transition of care in discharging an older adult, defined as surgical teams *‘assessing social support and need for home health before discharge, involving family and caregivers as appropriate in discharge planning, providing patient and caregiver with full list of medications and dosages, and emphasizing medication changes,’* is identified by the authors as a key element in quality care to reduce the risk of adverse events and hospital re-admission.

### Clinical and policy implications

Current guidance from the National Institute for Health and Care Excellence recommends ESD as a part of the post-operative hip fracture program for older adults who are medically stable, can transfer and mobilise short distances and have identifiable rehabilitation goals [35]. Guidelines published by the British Orthopaedic Association identify ESD as a potential intervention for the hip fracture population, noting that it is not suitable for all patients [36]. In the UK’s National Health Service, hip fractures are estimated to cost the service over £2 billion annually [37]. In a single arm intervention study carried out by Kapur, Thorpe [37], LoS was reduced by an average of 9.46 days when compared to data collected retrospectively from usual care prior to the ESD intervention commencing. With a population of 146 patients, this was equivalent to 1962.24 bed days saved and a subsequent saving of over £981,000.

As discussed previously, Closa, Mas [22] and Parsons, Parsons [24] were the two included studies to measure financial costs as an outcome. Both showed that reductions in the cost of care per patient are evident when an ESD model of care is provided to older adults when compared to usual care. From this, it can be hypothesised that a longer LoS is associated with increased resource costs, with similar findings in a systematic review carried out by Landeiro, Roberts [38].

### Areas for further research

ESD has the potential to reduce LoS post-operatively in an older adult population admitted to hospital with orthopaedic complaints. Future research should focus on high quality, multi-centre RCTs, that are sufficiently powered and adhering to CONSORT standardized reporting guidelines [39].

While the existing literature focuses on patient and process outcomes, little research has investigated the impact of ESD on stakeholders, including carer/family and the patient themselves. Hestevik, Molin [40] explored older adults experiences of their discharge from hospital to home. In this meta-summary of 13 qualitative studies, a core theme identified was that of patients feeling unsafe in their transition home, commonly due to a lack of communication. Participants reported that they felt their discharge was often rushed, with key information omitted, with decisions often being made for them by hospital staff. Similarly, Rodakowski, Rocco [41] carried out a systematic review and meta-analysis of 15 studies investigating caregiver integration in discharge planning. The authors found a 25% less risk of 90-day re-admission and 24% less risk of 180-day re-admission in older adults whose caregivers were involved in the discharge planning process when compared to usual care.

The studies included in this review were predominantly investigating ESD as an intervention in older adults who experience a traumatic hip fracture. However, the role of ESD in elective arthroplasty has not yet been explored. More recently, patients who are undergoing elective surgery for a total knee replacement or total hip replacement are experiencing shorter LoS [42]. A cohort study by Mundi, Axelrod [42] consisting of 333,212 adults (median age range 63–67), of whom almost one third returned home day one post-operatively. In the cohort who experienced an accelerated discharge, the risk of post-operative complications was no greater than those who experienced a LoS > one day. The authors did not disclose the rehabilitation provided to the patients; however future research should explore the role of ESD in tandem with an accelerated discharge programme.

### Strengths and weaknesses of the study

The use of PRISMA guidelines and robust methods are a strength of this systematic review and meta-analysis [14]. Various search engines were used with a broad search strategy as well as the use of a second and third reviewer to assess studies for inclusion further strengthen this review. A new quality assessment tool was employed by two authors again to ensure accuracy in assessing risk of bias following strict methods and guidance [19].

Limitations to this review include the high levels of heterogeneity in terms of the interventions provided and outcomes measured across the small number of included studies. Unpublished literature as well as non-English language papers were not included in the search strategy. A protocol was not published prior to this review being completed. Interventions provided varied in terms of the resources available, and the subsequent service provided to patients. The large variability in the outcomes measured and lack of standardisation makes the interpretation of the meta-analysis more difficult and may reduce the robustness of the meta-analysis [43]. Subgroup analysis and publication bias assessment was not possible due to the limited number of outcomes reported by the small number of studies included. Results may be interpreted with some caution.

## Conclusion

This systematic review and meta-analysis suggests that providing an ESD intervention to older adults admitted to hospital post orthopaedic surgery can significantly reduce their acute hospital LoS, without adversely affecting their function or the number of patients that experience falls. Future research should focus on older adults with general surgical complaints and those undergoing elective orthopaedic procedures using standardised interventions, outcome measures and be informed by the development of core outcome sets to reduce the levels of heterogeneity in the research. While ESD interventions are beneficial for this population group, the requirement for more defined and comprehensive interventions persists in conjunction with a detailed evaluation of the cost-effectiveness in practice.

### Electronic supplementary material

Below is the link to the electronic supplementary material.

Supplementary Material 1

Supplementary Material 2

Supplementary Material 3

Supplementary Material 4

Supplementary Material 5

Supplementary Material 6

## Data Availability

The datasets used and/or analysed during the current study available from the corresponding author on reasonable request.
