# Supplementary material for: Early supported discharge for older adults admitted to hospital after orthopaedic surgery: a systematic review and meta-analysis
Source: BMC Geriatr. 2024 Feb 9;24:143. doi: 10.1186/s12877-024-04775-y (PMC10858593; doi:10.1186/s12877-024-04775-y)
Supplement: Supplementary file 1 — Supplementary Material 1 [file 12877_2024_4775_MOESM1_ESM.docx]

**Supplementary File 6: Abbreviations**

ESD: Early Supported Discharge

LoS: Length of Stay

QoL: Quality of Life

MDT: Multi-disciplinary Team

ERAS: Enhanced Recovery After Surgery

PRISMA: Preferred Reporting Items for Systematic Reviews and Meta-Analysis

GRADE: Grading of Recommendations, Assessment, Development and Evaluation

RCT: Randomized Control Trial

GP: General Practitioner

FIM: Functional Independence Measure

MMSE: Mini-Mental State Examination

TUG: Timed Up and Go

FES: Falls Efficacy Scale

BBS: Berg Balance Scale

CSI: Caregiver Strain Index

MBI: Modified Barthel Index

CGA: Comprehensive Geriatric Assessment

BI: Barthel Index

SD: Standard Deviation

IRQ: Inter-quartile Range

MD: Mean Difference

SMD: Standardised Mean Difference

FEM: Fixed Effects Model

RR: Risk Ratio

DVT: Deep Vein Thrombosis

PE: Pulmonary Embolism

L: Low Risk of Bias

SC: Some Concerns in Risk of Bias

H: High Risk of Bias
