# Supplementary material for: Early supported discharge for older adults admitted to hospital after orthopaedic surgery: a systematic review and meta-analysis
Source: BMC Geriatr. 2024 Feb 9;24:143. doi: 10.1186/s12877-024-04775-y (PMC10858593; doi:10.1186/s12877-024-04775-y)
Supplement: Supplementary file 2 — Supplementary Material 2 [file 12877_2024_4775_MOESM2_ESM.docx]

**Additional File 1 – Search Strategies**

**CENTRAL Search Strategy**

#1 [mh ‘aged’]

#2 [mh ‘aging’]

#3 (late life OR elder* OR aged OR old age OR geriatric OR seniors): ti/ab/kw

#4 (old OR older OR aging OR aged OR senior OR elder*) NEAR/3 (person OR persons OR people OR adult* OR subject* OR patient* OR consumer* OR male* OR female* OR men OR women): ti/ab/kw

#5 (#1 OR # 2 OR #3 OR #4)

#6 [mh ‘patient discharge’]

#7 [mh ‘progressive patient care’]

#8 [mh ‘home care services’]

#9 [mh ‘home care services, hospital-based’]

#10 [mh ‘home nursing’]

#11 (early supported discharge OR ESD): ti/ab/kw

#12 ((early OR earlier OR prompt OR accelerate* OR acute OR subacute OR supported) NEAR/5 discharg*): ti/ab/kw

#13 (reduce* NEAR/5 (duration OR length) NEAR/5 (stay OR hospital)): ti/ab/kw

#14 (reduce* NEAR/5 (hospital OR inpatient OR in-patient) NEAR/5 (stay OR care)): ti/ab/kw

#15 ‘short-term ward’: ti/ab/kw

#16 ((organi?ed OR multidisciplinary) NEAR/5 discharge NEAR/5 team*): ti/ab/kw

#17 ((early OR earlier OR prompt OR accelerate* OR supported) NEAR/5 return* NEAR/2 home*): ti/ab/kw

#18 (hospital* NEAR/3 home*): ti/ab/kw

#19 ‘hospital rehabilitation unit*’: ti/ab/kw

#20 (rehabilitation near/3 home*): ti/ab/kw

#21 (intensive NEAR/2 home NEAR/5 (rehabilitation OR support*)): ti/ab/kw

#22 (mobile NEAR/2 team*): ti/ab/kw

#23 ((post-discharge OR home rehabilitation) NEAR/5 (support* OR care)): ti/ab/kw

#24 ((early OR earlier OR acute OR subacute OR post-discharge) NEAR/5 (community OR domiciliary OR primary care OR home OR home-based) NEAR/5 (rehabilitation OR support* OR care)): ti/ab/kw

#25 (#6 OR #7 OR #8 OR #9 OR #10 OR #11 OR #12 OR #13 OR #14 OR #15 OR #16 OR #17 OR #18 OR #19 OR #20 OR #21 OR #22 OR #23 OR #24)

#26 [mh ‘orthopedic procedure’]

#27 [mh ‘orthopedic procedures’]

#28 [mh ‘surgery’]

#29 [mh ‘operative surgical procedures’]

#30 [mh ‘elective surgical procedures’]

#31 [mh ‘orthopedic fixation devices’]

#32 [mh ‘orthopedic surgeons’]

#33 [mh ‘manipulation, orthopedic’]

#34 [mh ‘wounds and injuries’]

#35 (#26 OR #27 OR #28 OR #29 OR #30 OR #31 OR #32 OR #33 OR #34)

#36 (#5 AND #25 AND #35)

**MEDLINE Search Strategy**

1. MH ‘aged+’

2. MH ‘aging+’

3. (Late life OR elder* OR aged OR old age OR geriatric OR seniors) ti/ab

4. ((old OR older OR aging OR aged OR senior OR elder*) N3 (person OR persons OR people OR adult* OR subject* OR patient* OR consumer* OR male* OR female* OR men OR women)) ti/ab

5. (S1 OR S2 OR S3 OR S4)

6. MH ‘patient discharge’

7. MH ‘progressive patient care’

8. home care services OR home care services, hospital-based OR home nursing ti/ab

9. (early supported discharge OR ESD) ti/ab

10. ((early OR earlier OR prompt OR accelerate* OR acute OR subacute OR supported) N5 discharg*) ti/ab

11. (reduce* N5 (duration OR length) N5 (stay OR hospital)) ti/ab

12. (reduce* N5 (hospital OR inpatient OR in-patient) N5 (stay OR care)) ti/ab

13. ‘short-term ward’ ti/ab

14. ((organi?ed OR multidisciplinary) N5 discharge N5 team*) ti/ab

15. ((early OR earlier OR prompt OR accelerate$ OR supported) N5 return* N2 home*) ti/ab

16. (hospital* N3 home*) ti/ab

17. ‘hospital rehabilitation unit*’ ti/ab

18. (rehabilitation N3 home*) ti/ab

19. (intensive N2 home N5 (rehabilitation OR support*)) ti/ab

20. (mobile N2 team*) ti/ab

21. ‘organi?ed home care’ ti/ab

22. ((post-discharge OR home rehabilitation) N5 (support* OR care)) ti/ab

23. ((early OR earlier OR acute OR subacute OR post-discharge) N5 (community OR domiciliary OR primary care OR home OR home-based) N5 (rehabilitation OR support* OR care)) ti/ab

24. (S6 OR S7 OR S8 OR S9 OR S10 OR S11 OR S12 OR S13 OR S14 OR S15 OR S16 OR S17 OR S18 OR S19 OR S20 OR S21 OR S22 OR S23)

25. randomi?ed controlled trials ti/ab

26. random allocation ti/ab

27. controlled clinical trials ti/ab

28. control groups ti/ab

29. single-blind ti/ab

30. research design ti/ab

31. program evaluation ti/ab

32. random* ti/ab

33. (controlled N5 (trial* OR stud*)) ti/ab

34. (clinical* N5 trial*) ti/ab

35. ((control OR treatment OR experiment* OR intervention) N5 (group* OR subject* OR patient*)) ti/ab

36. (quasi-random* OR quasi random* OR pseudo-random* OR pseudo random*) ti/ab

37. ((control OR experiment* OR conservative) N5 (treatment OR therapy OR procedure OR manage*)) ti/ab

38. (assign* OR allocate*) ti/ab

39. controls ti/ab

40. trial ti/ab

41. (S25 OR S26 OR S27 OR S28 OR S29 OR S30 OR S31 OR S32 OR S33 OR S34 OR S35 OR S36 OR S37 OR S38 OR S39 OR S40)

42. MH ‘orthopedics’

43. MH ‘orthopedic procedures+’

44. MH ‘orthopedic surgeons’

45. MH ‘wounds and injuries’

46. MH ‘elective surgical procedures’

47. (‘orthopedic’ OR ‘orthopaedic’) ti/ab

48. (‘operation’ OR ‘operative’) ti/ab

49. MH ‘manipulation, orthopedic’

50. (S42 OR S43 OR S44 OR S5 OR S46 OR S47 OR S48 OR S49)

51. S5 AND S24 AND S41 AND S50

**CINAHL Search Strategy**

S1 (MH "Aged+")

S2 (MH "Aging+")

S3 (Late life OR elder* OR aged OR old age OR geriatric OR seniors) ti/ab

S4 ((old OR older OR aging OR aged OR senior OR elder*) N3 (person OR persons OR people OR adult* OR subject* OR patient* OR consumer* OR male* OR female* OR men OR women)) ti/ab

S5 S1 OR S2 OR S3 OR S4

S6 (MH "Home Health Care") OR (MH "Home Rehabilitation+") OR (MH "Home Nursing")

S7 ( TI ( (early OR earlier OR prompt OR accelerate* OR acute OR subacute OR supported) ) AND TI discharge* ) OR ( AB ( (early OR earlier OR prompt OR accelerate* OR acute OR subacute OR supported) ) AND AB discharge* )

S8 ( TI reduce* AND TI ( (duration OR length) ) AND TI ( (stay OR hospital) ) ) OR ( AB reduce* AND AB ( (duration OR length) ) AND AB ( (stay OR hospital) ) )

S9 ( TI reduc* AND TI ( (hospital OR inpatient OR in-patient) ) AND TI ( (stay OR care) ) ) OR ( AB reduc* AND AB ( (hospital OR inpatient OR inpatient) ) AND AB ( (stay OR care) ) )

S10 TI short-term ward OR AB short-term ward

S11 ( TI ( (organi?ed OR multidisciplinary) ) AND TI discharge AND TI team* ) OR ( AB ( (organi?ed OR multidisciplinary) ) AND AB discharge AND AB team* )

S12 ( TI ( (early OR earlier OR prompt OR accelerate* OR supported) ) AND TI return* AND TI home* ) OR ( AB ( (early OR earlier OR prompt OR accelerate* OR supported) ) AND AB return* AND AB home* )

S13 TI ( (hospital* AND home*) ) OR AB ( (hospital* AND home*) )

S14 TI hospital rehabilitation unit* OR AB hospital rehabilitation unit*

S15 TI ( (rehabilitation AND home*) ) OR AB ( (rehabilitation AND home*) )

S16 ( TI intensive AND TI home AND TI ( (rehabilitation OR support*) ) )OR ( AB intensive AND AB home AND AB ( (rehabilitation or support*) ) )

S17 TI ( (mobile AND team*) ) OR AB ( (mobile AND team*) )

S18 TI organi?ed home care OR AB organi?ed home care

S19 ( TI ( (post-discharge OR home rehabilitation) ) AND TI ( (support* OR care) ) ) OR ( AB ( (post-discharge OR home rehabilitation) ) AND AB ( (support* OR care) ) )

S20 ( TI ( (early OR earlier OR acute OR subacute OR post-discharge) ) AND TI ( (community OR domiciliary OR primary care OR home OR homebased) ) AND TI ( (rehabilitation OR support* OR care) ) ) OR ( AB ( (early OR earlier OR acute OR subacute OR post-discharge) ) AND AB ( (community OR domiciliary OR primary care OR home OR home-based) ) AND AB ( (rehabilitation OR support* OR care) ) )

S21 S6 OR S7 OR S8 OR S9 OR S10 OR S11 OR S12 OR S13 OR S14 OR S15 OR S16 OR S17 OR S18 OR S19 OR S20

S22 (MH "Randomized Controlled Trials") OR (MH "Random Assignment") OR (MH "Random Sample+")

S23 (MH "Clinical Trials") OR (MH "Intervention Trials") OR (MH "Therapeutic Trials")

S24 (MH "Single-Blind Studies")

S25 (MH "Control (Research)") OR (MH "Control Group") OR (MH "Placebos") OR (MH "Placebo Effect")

S26 (MH "Crossover Design") OR (MH "Quasi-Experimental Studies")

S27 PT (clinical trial OR randomized controlled trial)

S28 TI (random* OR RCT OR RCTs) OR AB (random* OR RCT OR RCTs)

S29 TI (controlled N5 (trial* OR stud*)) OR AB (controlled N5 (trial* OR stud*))

S30 TI (clinical* N5 trial*) OR AB (clinical* N5 trial*)

S31 TI ((control OR treatment OR experiment* OR intervention) N5 (group* OR subject* OR patient*)) OR AB ((control OR treatment OR experiment* OR intervention) N5 (group* OR subject* OR patient*))

S32 TI ((control OR experiment* OR conservative) N5 (treatment OR therapy OR procedure OR manage*)) OR AB ((control OR experiment* OR conservative) N5 (treatment OR therapy OR procedure OR manage*))

S33 TI (cross-over OR cross over OR crossover) or AB (cross-over OR cross over OR crossover)

S34 TI trial

S35 TI (assign* OR allocat*) OR AB (assign* OR allocat*)

S36 TI controls OR AB controls

S37 TI (quasi-random* OR quasi random* OR pseudo-random* OR pseudo random*) OR AB (quasi-random* OR quasi random* OR pseudorandom* OR pseudo random*)

S38 S22 OR S23 OR S24 OR S25 OR S26 OR S27 OR S28 OR S29 OR S30 OR S31 OR S32 OR S33 OR S34 OR S35 OR S36 OR S37

S39 (MH "orthopedic surgery+")

S40 (MH "manipulation, orthopedic")

S41 (MH "trauma+")

S42 (MH "wounds and injuries")

S43 (MH "surgery, elective+")

S44 (MH "surgery, operative+")

S45 TI (orthopedic OR orthopaedic) OR AB (orthopedic OR orthopaedic)

S46 TI (operation OR operative) OR AB (operation OR operative)

S47 S39 OR S40 OR S41 OR S42 OR S43 OR S44 OR S45 OR S46

S48 S5 AND S21 AND S38 AND S47

**EMBASE Search Strategy**

1. exp aged

2. exp aging

3. (Late life OR elder* OR aged OR old age OR geriatric OR seniors) ti/ab/kw

4. ((old OR older OR aging OR aged OR senior OR elder*) NEAR/3 (person OR persons OR people OR adult* OR subject* OR patient* OR consumer* OR male* OR female* OR men OR women)) ti/ab/kw

5. (#1 OR #2 OR #3 OR #4)

6. ‘hospital discharge’ ti/ab/kw

7. ‘early supported discharge’ ti/ab/kw

8. ‘progressive patient care’ ti/ab/kw

9. ‘home care’ OR ‘home physiotherapy’ OR ‘home rehabilitation’ ti/ab/kw

10. ‘home environment’ ti/ab/kw

11. ‘community based rehabilitation’ ti/ab/kw

12. (early supported discharge OR ESD) ti/ab/kw

13. ((early OR earlier OR prompt OR accelerate$ OR acute OR subacute OR supported) NEAR/5 discharg$) ti/ab/kw

14. (reduce$ NEAR/5 (duration OR length) NEAR/5 (stay OR hospital)) ti/ab/kw

15. (reduce$ NEAR/5 (hospital OR inpatient OR in-patient) NEAR/5 (stay OR care)) ti/ab/kw

16. short-term ward ti/ab/kw

17. ((organi?ed OR multidisciplinary) NEAR/5 discharge NEAR/5 team$) ti/ab/kw

18. ((early OR earlier OR prompt OR accelerate$ OR supported) NEAR/5 return$ NEAR/2 home$) ti/ab/kw

19. (hospital$ NEAR/3 home$) ti/ab/kw

20. hospital rehabilitation unit$ ti/ab/kw

21. (rehabilitation NEAR/3 home$) ti/ab/kw

22. (intensive NEAR/2 home NEAR/5 (rehabilitation OR support$)) ti/ab/kw

23. (mobile NEAR/2 team$) ti/ab/kw

24. organi?ed home care ti/ab/kw

25. ((post-discharge OR home rehabilitation) NEAR/5 (support$ OR care)) ti/ab/kw

26. ((early OR earlier OR acute OR subacute OR post-discharge) NEAR/5 (community OR domiciliary OR primary care OR home OR home-based) NEAR/5 (rehabilitation OR support$ OR care)) ti/ab/kw

27. #6 OR #7 OR #8 OR #9 OR #10 OR #11 OR #12 OR #13 OR #14 OR #15 OR #16 OR #17 OR #18 OR #19 OR #20 OR #21 OR #22 OR #23 OR #24 OR #25 OR #26

28. ‘Randomi?ed Controlled Trial’ ti/ab/kw

29. Randomi?ation ti/ab/kw

30. ‘Controlled Study’ ti/ab/kw

31. ‘control group’ ti/ab/kw

32. ‘Single Blind Procedure’ ti/ab/kw

33. ‘Parallel Design’ ti/ab/kw

34. random$ ti/ab/kw

35. (controlled NEAR/5 (trial$ OR stud$)) ti/ab/kw

36. ((control OR treatment OR experiment$ OR intervention) NEAR/5 (group$ OR subject$ OR patient$)) ti/ab/kw

37. ((control OR experiment$ OR conservative) NEAR/5 (treatment OR therapy OR procedure OR manage$)) ti/ab/kw

38. ((singl$) NEAR/5 (blind$ OR mask$)) ti/ab/kw

39. (assign$ OR alternate OR allocat$ OR counterbalance$ OR multiple baseline) ti/ab/kw

40. controls ti/ab/kw

41. trial ti/ab/kw

42. #28 OR #29 OR #30 OR #31 OR #32 OR #33 OR #34 OR #35 OR #36 OR #37 OR #38 OR #39 OR #40 OR #41

43. ‘surgery’ ti/ab/kw

44. ‘elective surgery’ ti/ab/kw

45. ‘injury’ ti/ab/kw

46. ‘orthopaedic surgery’ ti/ab/kw

47. ‘orthopedic surgery’ ti/ab/kw

48. ‘orthopaedic surgeon’ ti/ab/kw

49. ‘orthopedic surgeon’ ti/ab/kw

50. ‘emergency surgery’ ti/ab/kw

51. (orthopedic AND surge*) ti/ab/kw

52. (‘operation’ OR ‘operative’) ti/ab/kw

53. (‘orthopedic’ OR ‘orthopaedic’) ti/ab/kw

54. #43 OR #44 OR #45 OR #46 OR #47 OR #48 OR #49 OR #50 OR #51 OR #52 OR #53

55. #5 AND #27 AND #42 AND #54
