# Supplementary material for: Early supported discharge for older adults admitted to hospital after orthopaedic surgery: a systematic review and meta-analysis
Source: BMC Geriatr. 2024 Feb 9;24:143. doi: 10.1186/s12877-024-04775-y (PMC10858593; doi:10.1186/s12877-024-04775-y)
Supplement: Supplementary file 3 — Supplementary Material 3 [file 12877_2024_4775_MOESM3_ESM.docx]

**Additional File 3 – Cochrane Risk of Bias Tool**

| **Author (Year)** | **Domain 1** | **Domain 2** | **Domain 3** | **Domain 4** | **Domain 5** | **Overall** |
| --- | --- | --- | --- | --- | --- | --- |
| Berggren et al. (2019) | L | H | L | L | L | H |
| Closa et al. (2017) | H | L | L | SC | SC | H |
| Crotty et al. (2002) | L | L | L | L | L | L |
| Crotty et al. (2003) | L | L | L | L | L | L |
| Karlsson et al. (2016) | L | H | L | H | L | H |
| Karlsson et al. (2020) | L | H | L | H | L | H |
| Parsons et al. (2019) | L | SC | L | L | L | SC |

| Domain 1 | Risk of bias arising from randomisation process |
| --- | --- |
| Domain 2 | Risk of bias due to deviations from the intended interventions |
| Domain 3 | Missing outcome data |
| Domain 4 | Risk of bias in measurement of the outcome |
| Domain 5 | Risk of bias in the selection of the reported result |
| Overall RoB | Overall risk of bias |
