# Supplementary material for: Early supported discharge for older adults admitted to hospital after orthopaedic surgery: a systematic review and meta-analysis
Source: BMC Geriatr. 2024 Feb 9;24:143. doi: 10.1186/s12877-024-04775-y (PMC10858593; doi:10.1186/s12877-024-04775-y)
Supplement: Supplementary file 4 — Supplementary Material 4 [file 12877_2024_4775_MOESM4_ESM.docx]

**Additional File 4 – GRADE Assessment**

|  | Certainty Assessment | | | | | | | Summary of Findings | | | | |
| --- | --- | --- | --- | --- | --- | --- | --- | --- | --- | --- | --- | --- |
| Outcome | No of Studies | Study  Design | Risk of  Bias | Inconsistency | Indirectness | Imprecision | Other  Consider-ations | No of Patients | | Effect | | Certainty |
|  |  |  |  |  |  |  |  | ESD | Control | Relative  (95% CI) | Absolute (95% CI) |  |
| Length  of Stay | 4 | RCT's | serious^a^ | not serious | not serious | not serious | none | 433 | 608 | _ | MD 5.57  lower (7.07 lower to 4.08 lower) | Moderate |
|  | Comments: a. 4/4 studies were deemed to be of high risk of bias in terms of blinding of interventions. 1/4 studies used a geriatrician already working on the geriatric ward and familiar with the hospital who was unblinded to group allocation to register data collected. 1/4 studies allowed for participants to guide their randomisation. Given the inability for participants to be blinded to the intervention they receive and the potential role of bias in using a geriatrician familiar with the site the risk of bias was downgraded accordingly. | | | | | | | | | | | |
| Function | 3 | RCT's | serious^b^ | serious^c^ | not serious | serious^d^ | none | 232 | 406 | _ | MD 0.02  (1.13 lower to 1.08 higher) | Low |
|  | Comments:  b. 3/3 studies were deemed to be of high risk of bias in terms of blinding of interventions. Given the inability for participants to be blinded to the intervention they receive, the risk of bias was downgraded accordingly. c. Inconsistency was suitably downgraded as P = 0.93 and subgroup analysis was not possible, therefore remained unexplained. d. Imprecision was downgraded according as as result of wide confidence intervals in the forest plot | | | | | | | | | | | |
| Re-admissions | 2 | RCT's | serious^e^ | serious^f^ | serious^f^ | not serious | none | Narrative: Re-admission rates were similar in the  intervention and control groups when measured at four and twelve months respectively by 2 studies. | | | | Very Low |
|  | Comments: e. 2/2 studies were deemed to be of high risk of bias in terms of blinding of interventions. 1/2 studies used a geriatrician already working on the geriatric ward and familiar with the hospital who was unblinded to group allocation to register data collected. Given the inability for participants to be blinded to the intervention they receive and the potential role of bias in using a geriatrician familiar with the site the risk of bias was downgraded accordingly f. 2/2 trials had a population of older adults post surgery for traumatic hip fracture | | | | | | | | | | | |
| No of fallers | 2 | RCT's | serious^g^ | serious^h^ | serious^i^ | serious^j^ | none | 52/141 (36.9%) | 42/150 (32.3%) | RR 1.14 (0.83 to 1.57) | 45 more per 1,000 (from 55  fewer to 184 more) | Very Low |
|  | Comments: g. 2/2 studies were deemed to be of high risk of bias in terms of blinding of interventions. 2/2 studies used a geriatrician already working on the geriatric ward and familiar with the hospital who was unblinded to group allocation to register data collected. Given the inability for participants to be blinded to the intervention they receive and the potential role of bias in using a geriatrician familiar with the site the risk of bias was dow... h. Inconsistency was downgraded as P = 0.43 and for the width of the 95% CI i. 2/2 trials had a population of older adults post surgery for traumatic hip fracture, indirectness was downgraded appropriately. j. Wide confidence intervals in the forest plot resulted in imprecision being downgraded accordingly | | | | | | | | | | | |
| Carer  Satisfaction | 2 | RCT's | serious^k^ | serious^l^ | Serious^l^ | not serious | none | Narrative: Carer self-reported QoL was measured using the Short Form-36 in one study, with statistically significant effects favouring the intervention group seen at four months (MD = 0.01, 95% CI –13.8 to 13.8), but not at twelve months. | | | | Very Low |
|  | Comments: k. 2/2 trials had a population of older adults post surgery for traumatic hip fracture. l. 2/2 studies were deemed to be of high risk of bias in terms of blinding of interventions. . Given the inability for participants to be blinded to the intervention they receive the risk of bias was downgraded accordingly | | | | | | | | | | | |
