# Supplementary material for: Early supported discharge for older adults admitted to hospital after orthopaedic surgery: a systematic review and meta-analysis
Source: BMC Geriatr. 2024 Feb 9;24:143. doi: 10.1186/s12877-024-04775-y (PMC10858593; doi:10.1186/s12877-024-04775-y)
Supplement: Supplementary file 6 — Supplementary Material 6 [file 12877_2024_4775_MOESM6_ESM.docx]

**Additional File 5 – Forest Plots for Secondary Outcomes**


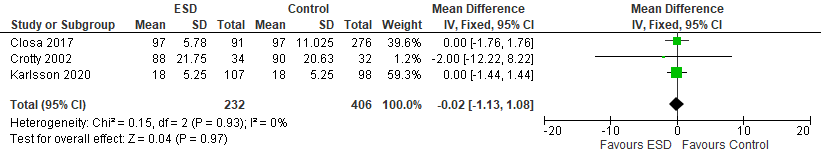


*Figure S1: Forest plot for function*


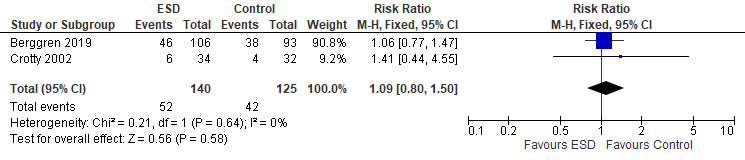


*Figure S2: Forest plot for number of fallers*
